# Molecular mechanisms of *N*-1-naphthylphthalamic acid, a chemical tool in plant biology and agriculture

**DOI:** 10.1186/s43897-022-00043-y

**Published:** 2022-09-26

**Authors:** Mengjuan Kong, Xin Liu, Linfeng Sun, Shutang Tan

**Affiliations:** MOE Key Laboratory for Cellular Dynamics, and School of Life Sciences, Division of Life Sciences and Medicine, University of Science and Technology of China, Hefei, 230027 China

The phytohormone auxin plays essential roles in modulating plant growth and development by regulating cell expansion, division, and differentiation. Genetic or pharmacological interference of the auxin pathway affects multiple growth and patterning processes in plants (Vanneste and Friml 2009). The research on auxin can be tracked to the earliest observations on plant growth regulation and phototropism by Sachs and Darwin in the nineteenth century (Friml 2022). Indole-3-acetic acid (IAA) is the major natural auxin form, and decades of genetic and biochemical studies have established the molecular framework for auxin biosynthesis, signaling, and transport (Friml 2022). Synthetic auxins (such as 2,4-D and dicamba) and anti-auxin compounds (such as *N*-1-naphthylphthalamic acid, NPA) have been widely used in modern agriculture and horticulture for decades. For example, synthetic auxins can be used for delaying fruit and leaf abscission, inducing parthenocarpic fruit, and promoting rooting of cut stems for plant propagation in many agricultural, horticultural, and forestry plants. Moreover, both synthetic auxins and anti-auxin chemicals are commercial herbicides. Therefore, development of novel chemical tools targeting the auxin pathway will be useful in both agriculture and horticulture.

One intriguing aspect of auxin is its directional intercellular transport, namely polar auxin transport (PAT), which is determined by polar plasma membrane (PM)-resident PIN-FORMED (PIN) transporters (Adamowski and Friml 2015). Decades of studies support that polar auxin transport generates spatial-temporarily dynamic auxin maxima and minima, ensuring both robustness and plasticity of developmental programming. Notably, amongst many other developmental processes, auxin transport plays vital roles in regulating shoot branching and floral development. Breaking apical dominance by removal of the shoot apex or by application of auxin transport inhibitors leads to outgrowth of axillary buds into branches. This phenomenon has long been noticed and it is often employed in various agricultural, horticultural, and forestry plants to shape the shoot architecture, which is closely related to the yield. Indeed, the shoot architecture is an important trait for domestication of many crops (such as rice and maize) and horticultural plants (such as apples) (Barbier et al. 2017). In auxin research, the synthetic compound NPA has been used as an auxin transport inhibitor since 1950s (Teale and Palme 2018). Recent biochemical and structural studies reveal that NPA directly associates with and inhibits PIN auxin transporters (Abas et al. 2021; Teale et al. 2021; Lam Ung et al. 2022; Su et al. 2022; Yang et al. 2022). Nonetheless, apart from the direct NPA-PIN interaction model, multiple working hypotheses were once proposed to explain the action mode of NPA. Herein we focus on discussing these recent advances on molecular mechanisms underlying NPA inhibiting auxin transport.

Before the molecular cloning of *PIN* family transporter genes (Gälweiler et al. 1998; Luschnig et al. 1998), pharmacological interference of auxin transport, including NPA, has long been used in auxin research. As early as in 1950s, researchers discovered that synthetic transport inhibitors could be used to analyze the PAT function in plants (Teale and Palme 2018). According to their origin, auxin transport inhibitors can be divided into endogenous plant substances (flavonoids, tannins, coumarins, etc.), native compounds of non-plant origin (Brefeldin A) and fully synthetic drugs (NPA; 2,3,5-Triiodobenzoic acid, TIBA; etc.). Amongst others, NPA is widely used in plant auxin research, and it can also be used as an herbicide in agriculture (Teale and Palme 2018). NPA is a small molecule compound with a similar chemical structure to IAA (Fig. 1A). The fact of NPA affecting plant inflorescence development and tropic growth suggests that it is an inhibitor of PAT. Further transport assays support a specific inhibitory effect of NPA on auxin export. Indeed, NPA has a variety of physiological effects on embryo development, lateral root development, apical dominance, adventitious root formation, etc. Though without biochemical determination of the *in planta* target in the early studies, NPA has been widely used in both biological studies and agricultural applications. It is a pivotal tool in elucidating the unique PAT processes underlying plant growth and development. Multiple protein targets were proposed to mediate NPA function *in planta*: B-type ATP-binding cassette (ABC) transporters, TWISTED DWARF1 (TWD1, also called FKBP42), Aminopeptidase 1 (APM1), and PIN proteins. These non-PIN proteins as NPA targets were discussed previously (see review by Teale and Palme 2018).

**Fig. 1:**
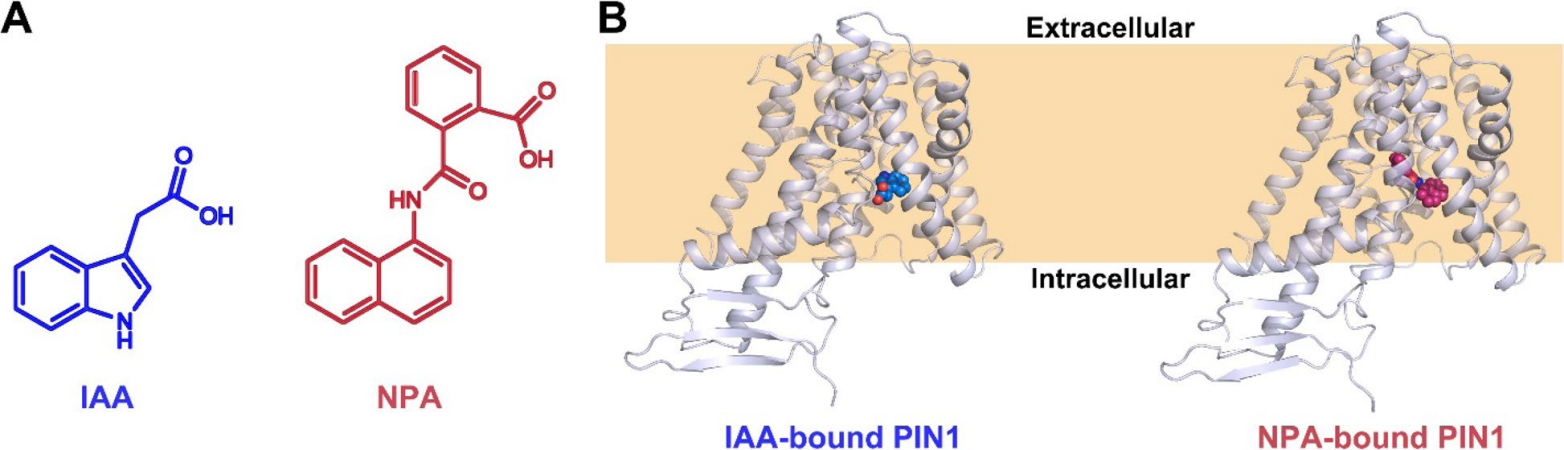
Structures of auxin- and NPA-bound PIN1 protein. **A** Chemical structures of the natural auxin IAA and the synthetic inhibitor NPA. **B** Structures of IAA- and NPA-bound PIN1 protein. Left, IAA-bound PIN1 structure (PDB accession code: 7Y9V). Right, NPA-bound PIN1 structure (PDB accession code: 7Y9U, Yang et al. 2022). PIN1 protein is shown in a cartoon mode and colored light blue. IAA and NPA molecules are shown in spheres, with carbon atoms colored blue and red, respectively

PIN family proteins are responsible for exporting auxin from the cytoplasm to apoplast in plants. PM-resident PIN proteins are always polarly-distributed, varying in different cell types. An important question is the relation between PIN polar localization and auxin flow direction, which indicates that the asymmetric distribution of auxin in plants (Adamowski and Friml 2015). There are eight members of the PIN protein family (PIN1-PIN8) in *Arabidopsis*. PIN proteins have similar structures: amino- and carboxy-terminal conserved membrane-spanning domains and a central divergent hydrophilic domain (hydrophilic loop). Based on sequence similarities and the length of the hydrophilic loop, PIN proteins can be divided into three categories: PIN1-PIN4 and PIN7 (long PINs), PIN5 and PIN8 (short PINs), and PIN6 (intermediate PIN). Different PIN subclades have evolved specific biological functions. In general, long PINs localize to the PM and exhibit asymmetric distribution, which determines the direction of intercellular auxin flow. PIN5 and PIN8 localize to the endoplasmic reticulum and regulate auxin homeostasis in cells by regulating auxin flow between the cytoplasm and the endoplasmic reticulum. There is a high degree of functional redundancy amongst *PIN* genes (Adamowski and Friml 2015).

When looking back to the physiological effects of NPA, it highly phenocopies *pin* loss-of-function (single or multiple) mutants, in terms of root gravitropism, lateral root formation, shoot phototropism, inflorescence development, vascular patterning, etc. Okada et al. found that *Arabidopsis pin1* mutants have naked pin-like inflorescence, abnormal flowers and leaves, which is reminiscent of NPA-treated plants (Okada et al. 1991), indicating a strong connection between NPA and PIN1. Though NPA is widely used in plant biology as an inhibitor specifically suppressing auxin efflux and thus polar auxin transport across cells, the molecular mechanisms became clear only recently. Teale et al. found that NPA can directly inhibit PIN1-mediated auxin transport by using an auxin responsive reporter co-expressed with PIN1 in *Arabidopsis* leaf protoplasts. A parallel study by Abas et al. showed that NPA could bind to PIN proteins on plasma membrane and thus inhibit auxin transport, indicating that PIN is the target protein of NPA.

Recently, three research teams independently reported the structures of *Arabidopsis* PIN1 (Yang et al. 2022), PIN3 (Su et al. 2022), and PIN8 (Lam Ung et al. 2022) in different states (apo-, IAA-bound, and NPA-bound, respectively). Notably, these structural studies have advanced our understanding on the auxin transport mechanisms of PIN proteins as well as their inhibition by NPA. Based on the structures, both the apo-form and IAA-bound PIN1 or PIN3 proteins exhibit an almost identical inward-facing conformation (Fig. 1B), while PIN8 exhibit an outward-facing conformation in the apo-form or IAA-bound state. For PIN1, PIN3, and PIN8, all the NPA-bound structures show an inward-facing state (Fig. 1B). These results indicate that NPA binds directly to PIN proteins, but not through other PIN regulators indirectly, locking PIN proteins in an inward-facing state that is unable to undergo conformational changes for IAA transport. These structural studies further confirmed that PIN auxin transporters are the real targets of NPA, and also elucidated the underlying mechanisms.

Collectively, multiple lines of genetic, biochemical, and structural evidence strongly support that PIN auxin transporters are bona fide targets of NPA *in planta*. This is crucial for the interpretation of previous and future NPA-involving experiments. Nonetheless, certain cellular effects of NPA, such as regulation of actin cytoskeleton dynamics and endomembrane trafficking, may not be fully elucidated by PINs. These effects might be mediated by additional targets or simply indirect effects of an intracellular auxin increase. Unraveling the underlying molecular mechanisms will be helpful to guide its use in both plant research and agricultural application. Moreover, future work on the genetic engineering of endogenous *PIN* family genes or design of highly selective PIN inhibitors might be useful in both agriculture and horticulture.

## Data Availability

Not applicable.
